# Introducing Fast Fourier Convolutions into Anomaly Detection

**DOI:** 10.3390/s25165196

**Published:** 2025-08-21

**Authors:** Zhen Zhao, Jiali Zhou

**Affiliations:** 1 College of Intelligent Manufacturing, Zhejiang Polytechnic University of Mechanical and Electrical Engineering, Hangzhou 310053, China; zhaozhen@zime.edu.cn; 2 Institute of Mathematics and AI Applications, Zhejiang University of Technology, Hangzhou 310023, China

**Keywords:** anomaly detection, deep learning, industry applications

## Abstract

Anomaly detection is inherently challenging, as anomalies typically emerge only at test time. While reconstruction-based methods are popular, their reliance on CNN backbones with local receptive fields limits discrimination and precise localization. We propose FFC-AD, a reconstruction framework using Fourier Feature Convolutions (FFCs) to capture global information early, and we introduce Hidden Space Anomaly Simulation (HSAS), a latent-space regularization strategy that mitigates overgeneralization. Experiments on MVTec AD and VisA demonstrate that FFC-AD significantly outperforms state-of-the-art methods in both detection and segmentation accuracy.

## 1. Introduction

Anomaly detection aims to identify outliers that deviate significantly from normal patterns and precisely localize anomalous regions, with critical applications in industrial quality inspection, medical diagnosis, and video surveillance [1,2,3]. The rarity and diversity of anomalies—such as micro-cracks in semiconductor wafers—pose major challenges: undetected defects can increase rework rates and reduce profit margins by 20–40% [4]. To combat this, manufacturers are increasingly adopting AI-driven inspection systems; for instance, Omron’s AI solution achieves real-time detection and improves yield rates by 25% over manual inspection [5]. Since anomalies are inherently scarce, most datasets include defective samples only during testing, further complicating model training. These practical demands have driven growing interest in anomaly detection from both academia and industry.

Anomaly detection methods are commonly categorized as embedding-based or reconstruction-based. Embedding-based methods rely on pre-trained models (e.g., ImageNet [6]) to extract features but often suffer from distribution mismatch with target domains. Recent advances favor reconstruction-based approaches, where autoencoders learn the distribution of normal samples through encoding and decoding. However, two major limitations remain: (1) CNN-based architectures have limited receptive fields, restricting global context modeling; (2) overly general autoencoders may produce “identity mappings” [7], reconstructing anomalies as normal and impairing detection.

To address these limitations, we propose Fourier Feature Convolution Anomaly Detection (FFC-AD), a reconstruction-based framework that enhances global context modeling and mitigates overgeneralization. In FFC-AD, standard convolutional layers in both the encoder and decoder are replaced with Fourier Feature Convolutions (FFCs) [8], which operate in the frequency domain to enable global information capture even in shallow layers [9]. To further improve anomaly discrimination, we introduce Hidden Space Anomaly Simulation (HSAS), a latent-space regularization strategy that injects dropout-based perturbations during training to simulate defects [7,10]. This prevents invariant reconstructions [11] and reduces overgeneralization, improving the model’s ability to distinguish unseen anomalies. Our architecture comprises a frozen FFC encoder, an FFC-based denoising autoencoder, and a segmentation head. The encoder adopts a ResNet-like design [12], with each FFC block incorporating local and global branches for context-aware representation learning. The reconstruction network aligns hierarchical features from the decoder with those from the encoder, ensuring consistent restoration of normal patterns while maintaining robustness through frequency-domain priors.

Our contributions are summarized as follows:

- We introduce Fourier Feature Convolutions (FFCs) into anomaly detection to enable early-stage global context modeling and improve reconstruction quality.
- We propose Hidden Space Anomaly Simulation (HSAS), a latent-space regularization strategy that mitigates overgeneralization and enhances anomaly discrimination.
- We design a dual-branch FFC architecture that aligns hierarchical local–global features between encoder and decoder, improving the consistency of normal reconstructions.
- Our method achieves state-of-the-art pixel-wise AP on MVTec AD (77.0%) and VisA (46.6%), demonstrating strong performance in both detection and localization tasks.

The remainder of this paper is structured as follows: In Section 2, we conduct a thorough review of the existing literature, highlighting the research gaps that our work aims to address. In Section 3, we describe the methodology employed in our study, including detailed information about the experimental setup and the architecture of our proposed model. The results of our experiments are presented in Section 4, where we provide a comprehensive analysis of the findings, compare them with those from prior studies, and explore the underlying reasons for any observed differences. In Section 5, we delve deeper into several critical aspects of our proposed framework, exploring its potential extensions, limitations, and future research directions. Lastly, in Section 6, we summarize the main conclusions drawn from our research and discuss the broader implications of our findings for the field.

## 2. Related Works

**Anomaly Detection.** The existing methodologies for anomaly detection can be broadly categorized into two main types: embedding-based and reconstruction-based approaches.

Embedding-based methods exploit pre-trained models to extract robust feature representations and discern anomalies within a high-dimensional space. CFA [13] introduces a learnable patch descriptor that embeds target-oriented features, alongside a scalable memory bank independent of the dataset size. This method employs transfer learning to determine the center and surface of the hypersphere in the memory bank using the positional relationship between the test feature and this coupled hypersphere to identify anomalies. PatchCore [14] operationalizes this concept by executing k-nearest neighbor searches during the testing phase, where the distance between the test feature and normal features in the memory bank serves as an anomaly score. CFlow [15] incorporates positional encoding into the conditional normalized flow framework, leading to improved outcomes. PyramidFlow [16], with its invertible pyramids and pyramid coupling blocks, facilitates multi-scale fusion and mapping, thereby enabling precise high-resolution anomaly localization.

Reconstruction-based techniques utilize encoder–decoder architectures to pinpoint anomalies by analyzing reconstruction errors. DRAEM [10] simulates defects using Perlin noise, training the network to accurately localize defects by segmenting defect masks. RD [17] adopts knowledge distillation from a pre-trained teacher network to a student network; during inference, discrepancies between the teacher’s faithful feature extraction and the student’s anomaly-free outputs signify abnormalities. SimpleNet [11] simulates defect distributions via Gaussian noise and distinguishes between normal and abnormal samples using a discriminator. UniAD [7] employs a Transformer-based reconstruction network to train the model in reconstructing defective samples as normal ones. RD++ [18] integrates projection layers after each intermediate teacher block to provide compact, anomaly-free representations to the student network. DeSTSeg [19] reconstructs normal samples at the feature level and precisely locates defects through a segmentation network. AnomalyDiffusion [20] classifies anomalies into appearance and location categories to generate diverse and realistic anomaly images using a diffusion network. RealNet [21], a feature reconstruction network, effectively leverages multi-scale pre-trained features for anomaly detection by adaptively selecting pre-trained features and reconstruction residuals. DiAD [20] fine-tunes Stable Diffusion to enhance pixel-level image-to-image translation capabilities on defective samples, enabling consistent semantic preservation while reconstructing anomalies. DDAD [22] adopts a conditioning-based denoising strategy, where the input image serves as a guidance target throughout the denoising process. Additionally, domain adaptation is introduced to improve the effectiveness of feature-wise comparisons across domains. GLAD [23] proposes a global adaptive mechanism tailored to each input sample, allowing the model to retain as much normal information as possible. This design introduces greater flexibility and enables anomaly-free reconstructions with minimal semantic drift. ViTAD [24] presents a Vision-Transformer-based framework that systematically incorporates both global and local representations for improved anomaly detection, demonstrating the effectiveness of hierarchical design in Transformer-based models.

**Fourier Transform.** The Fourier Transform has been a fundamental tool in digital image processing for decades [25]. Currently, numerous studies have begun to integrate the Fourier Transform into deep learning methods to enhance performance. FDA [26] leverages operations in the frequency domain to achieve domain adaptation and alignment. GFN [27] learns long-term spatial dependencies by replacing self-attention with a 2D discrete Fourier transform, an element-wise multiplication between frequency-domain features and learnable global filters, and a 2D inverse Fourier transform, all performed with log-linear complexity. FNO [28] conducts full matrix multiplication across all channels after applying the Fourier transform. AFNO [29] imposes a block-diagonal structure on the channel mixing weights, adaptively sharing weights across tokens and sparsifying the frequency modes via soft-thresholding and shrinkage. FourierAE [30] integrates Fourier Transforms into Autoencoders and Variational Autoencoders, showcasing that frequency-domain features can provide less noisy representations for anomaly detection. The authors of [31] employ a Fourier space supervision loss to enhance the restoration of missing high-frequency content from the ground-truth image. Additionally, they design a discriminator architecture that operates directly in the Fourier domain, aiming to better match the target high-frequency distribution. FECNet [32] revisits the frequency properties of images with different exposure through the Fourier transform. It is found that the amplitude component contains most of the lightness information, and the phase component is related to the structure information. The network interactively processes the local spatial features and the global frequency information to promote complementary learning. DeepRFT [33] attempts to utilize kernel-level information for image deblurring networks. It applies the Fourier transform to the standard Res Block, enabling the exploitation of both kernel-level and pixel-level features by learning frequency–spatial dual-domain representations. AFSC [34] introduces an adaptive Fourier space compression method for anomaly detection. This method sparsely samples Fourier coefficients to retain global image information for normal reconstruction while discarding anomalies, achieving competitive results on industrial benchmarks without relying on external priors. FourierMamba [35] performs image deraining in the Fourier space. It applies zigzag coding to reorder frequencies for correlation and uses Mamba in the channel dimension to improve frequency-based information representation. These works collectively highlight the unique advantages of the Fourier Transform as a powerful mathematical tool for handling image data. By integrating the Fourier Transform with modern deep learning frameworks, researchers can not only explore new application scenarios but also provide more efficient solutions to existing problems.

## 3. Methodology

In this section, we first briefly formulate the popular reconstruction-based anomaly detection framework. Subsequently, we introduce how Fast Fourier Convolution (FFC) can be applied to anomaly detection tasks. Moreover, we present a novel anomaly simulation strategy to enhance the robustness of the model.

### 3.1. Preliminary

Reconstruction-based methods take an arbitrary image X from the training set as input, with the objective of training the network to reconstruct this image $\hat{X}$ as accurately as possible. The objective loss function can be formulated as follows:

(1) $$L_{recon}={∥X-\hat{X}∥}_{2}^{2}$$

The underlying The underlying premise is that since the reconstruction network is trained exclusively on anomaly-free samples, it struggles to accurately reconstruct defective samples. Thus, during the testing phase, comparing the network’s input X and output $\hat{X}$ enables the localization of anomalies, as denoted by the formula above.

Recently, some methodologies have proposed employing data augmentation techniques to simulate anomalies, thereby addressing issues such as overgeneralization or “identity mapping” [7]. In these cases, the network’s objective function encourages diversity in the reconstruction process:

(2) $$L_{recon}={∥X^{\prime }-\hat{X}∥}_{2}^{2}$$

where $X^{\prime }$ represents the simulated anomaly samples generated via carefully designed data augmentation strategies.

### 3.2. Framework

**Figure 1:**
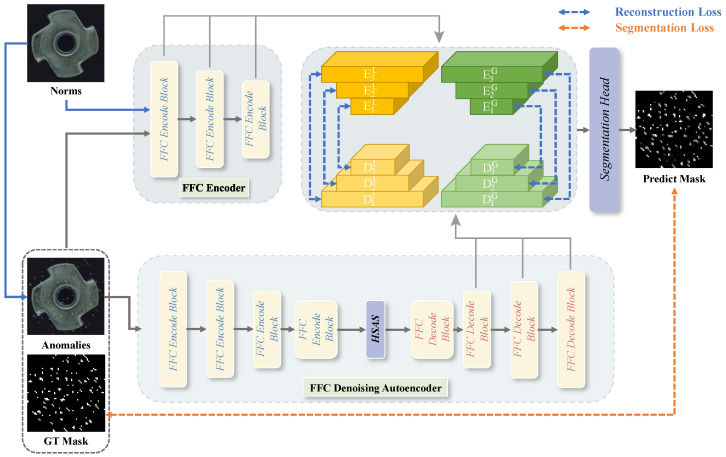
Overview of our FFC-AD framework, which consists of two primary components: the FFC encode block and the FFC decode block. During the reconstruction phase, the frozen FFC encoder processes normal images as input, while the FFC denoising autoencoder reconstructs simulated anomaly samples under the supervision of multi-level local/global features extracted from the FFC encoder. In the segmentation phase, the frozen FFC encoder processes the same simulated anomaly samples as the trained FFC denoising autoencoder, and the segmentation head precisely localizes the anomalies using a simulated ground-truth mask for supervision.

As illustrated in Figure 1, our FFC-AD framework comprises a frozen FFC encoder, an FFC denoising autoencoder that reconstructs anomalies into normal representations, and a segmentation head that distinguishes multi-level local/global FFC features to output the predicted segmentation mask. The FFC encoder and the FFC denoising autoencoder are constructed using FFC encoder blocks and FFC decoder blocks. The FFC encoder block follows the ResNet-18 design [12], while the FFC decoder block replaces all downsampling layers with upsampling layers. The training process of the proposed FFC-AD consists of two stages: training the FFC denoising autoencoder and training the segmentation head. In the first stage, the FFC encoder takes normal images as input, whereas the FFC denoising autoencoder receives images with anomalies generated via DRAEM [10]. Multi-level local/global features extracted from these inputs are aligned using reconstruction loss. Once the FFC denoising autoencoder acquires the ability to reconstruct normal images from anomalous ones, we proceed to train the segmentation head for precise anomaly localization. This is achieved by feeding normal images into the FFC encoder and anomalous images into the FFC denoising autoencoder. During the testing phase, anomaly samples are fed into both the FFC encoder and the FFC denoising autoencoder, and the output prediction mask serves as the predicted segmentation result. The final anomaly detection result is obtained by taking the maximum value in the mask.

### 3.3. Local/Global Context Perception

Existing convolutional neural networks (CNNs) typically adopt a chain-like topology to capture information at multiple scales. We observe that global information is generally only accessible in the deeper layers of the network. For high-level tasks, such as image classification, this design is acceptable because the output provides an abstract summary of all available information. However, low-level tasks usually require the output to maintain a similar spatial resolution and channel dimension as the input. Unlike high-level vision tasks, which focus on label accuracy, low-level vision tasks demand precise and clean pixel-level modeling, especially for image reconstruction tasks.

Due to the presence of large anomalies in the input images, the early layers with smaller receptive fields may entirely fall within these anomalies, thereby extracting only missing or corrupted information. This type of information is not useful for reconstructing anomaly-free targets and can even shift the overall bias as it propagates through subsequent layers. Therefore, we argue that incorporating a wide receptive field as early as possible in the framework would significantly assist smaller receptive fields in achieving near-perfect reconstruction, especially when dealing with large anomaly regions. Traditional deep convolutional network architectures [12] suffer from a slowly increasing effective receptive field, as a large number of early and shallow small 3×3 convolutions lack global context information. This issue becomes particularly pronounced for high-resolution images.

Fast Fourier Convolution (FFC) [8] aims to introduce global information early in the network while retaining the inherent local perception capabilities of CNNs. Given a feature map $F\in R^{H\times W\times C}$, FFC divides the input features into local and global branches. The local part $F^{l}\in R^{H\times W\times (1-\alpha )C}$ is designed to learn from the local neighborhood, while a second global part $F^{g}\in R^{H\times W\times \alpha C}$ captures long-range context. The parameter α∈[0,1] represents the percentage of feature channels allocated to the global part.

FFC mainly captures the global features of the input feature map through the Fourier Transform. The Fourier Transform is a fundamental mathematical tool that decomposes a function into its constituent frequencies. Within the realm of computer vision, the 2D Discrete Fourier Transform (DFT) is particularly useful for converting spatial features into their frequency-domain representations. Conversely, the 2D Inverse Discrete Fourier Transform (iDFT) facilitates the reconstruction of these frequency-domain features back into the spatial domain. The 2D Discrete Fourier Transform (DFT) and its inverse (iDFT) can be expressed as follows:

(3) $$F\left[k,l\right]=\sum_{m=0}^{M-1}\sum_{n=0}^{N-1}f\left[m,n\right]e^{-j2\pi \left(\frac{km}{M}+\frac{ln}{N}\right)}$$

(4) $$f\left[m,n\right]=\frac{1}{MN}\sum_{k=0}^{M-1}\sum_{l=0}^{N-1}F\left[k,l\right]e^{j2\pi \left(\frac{km}{M}+\frac{ln}{N}\right)}$$

where *m* and *n* are the spatial coordinates, and *k* and *l* are the frequency coordinates, respectively. $e^{-j2\pi \left(\frac{km}{M}+\frac{ln}{N}\right)}$ and $e^{j2\pi \left(\frac{km}{M}+\frac{ln}{N}\right)}$ are complex exponentials used for phase modulation in the forward and inverse transforms, respectively.

**Figure 2:**
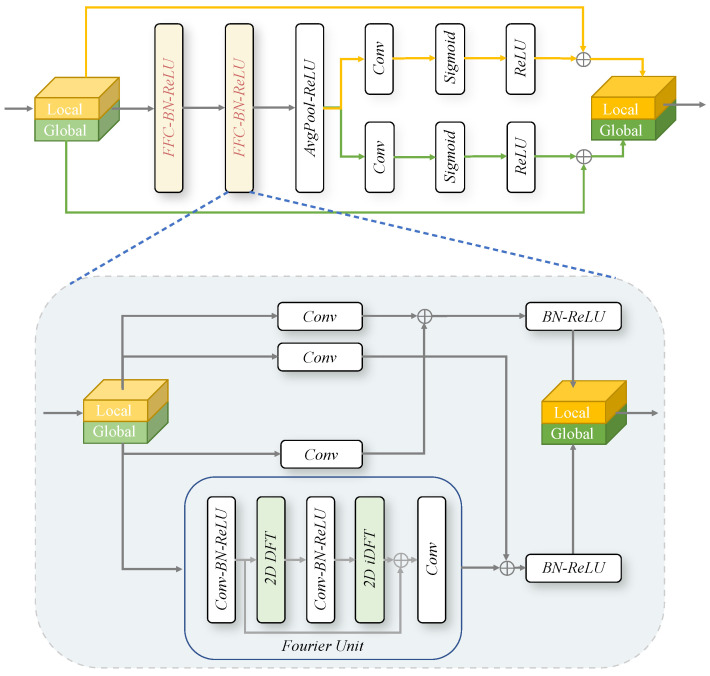
Structure of the FFC encoder block. The block primarily consists of two FFC layers for feature extraction, enhanced by a residual connection to mitigate vanishing gradient issues. Given an input feature map, the FFC layer separates the features into local and global branches. The local branch focuses on learning from neighboring regions, while the global branch captures long-range dependencies via the Fourier Transform. The Fourier Transform, a foundational mathematical tool, decomposes functions into their frequency components. In our approach, we employ the 2D Discrete Fourier Transform (DFT) to convert spatial features into frequency-domain representations and the 2D Inverse Discrete Fourier Transform (iDFT) to reconstruct these features back into the spatial domain.

As illustrated in Figure 2, the FFC module initially partitions the feature maps into two branches: a local branch and a global branch. The local branch uses conventional convolution to extract local details, whereas the global branch leverages the Fourier Transform to obtain the global context. The steps involved in the Fourier Unit are as follows:

(a) applies a convolution block in the spatial domain:
  (5) $$ReLU\left(BN\left(Conv\right)\right):R^{H\times W\times C}\to R^{H\times W\times C};$$
(b) applies the 2D DFT to the global branch tensor and concatenates real and imaginary parts:
  (6) $$DFT:R^{H\times W\times C}\to C^{H\times \frac{W}{2}\times C},$$
  (7) $$ComplexToReal:C^{H\times \frac{W}{2}\times C}\to R^{H\times \frac{W}{2}\times 2C};$$
(c) applies a convolution block in the frequency domain:
  (8) $$ReLU\left(BN\left(Conv\right)\right):R^{H\times \frac{W}{2}\times 2C}\to R^{H\times \frac{W}{2}\times 2C};$$
(d) applies the 2D iDFT to recover its spatial structure:
  (9) $$RealToComplex:R^{H\times \frac{W}{2}\times 2C}\to C^{H\times \frac{W}{2}\times C},$$
  (10) $$iDFT:R^{H\times \frac{W}{2}\times C}\to R^{H\times W\times C};$$
(e) applies a residual connection process:
  (11) $$Connect:R^{H\times W\times C}\to R^{H\times W\times C}.$$

The feature map $F^{g\to g}$, after passing through the Fourier Unit, is combined with the local branch feature map $F^{l\to g}$ via concatenation to form the final global branch feature. Similarly, the final local branch feature is obtained by concatenating the features $F^{l\to l}$ and $F^{g\to l}$ from both the local and global branches.

Anomaly detection requires modeling both local fine-grained details and global structural coherence, as anomalies often disrupt either or both. FFC addresses this via dual paths: a spatial path capturing local patterns through standard convolutions and a spectral path modeling global context in the frequency domain. The frequency domain naturally encodes global structure (via low-frequency components) and local variations (high frequencies), with normal patterns exhibiting stable frequency statistics. Anomalies manifest as statistically significant deviations in these distributions (e.g., disrupted low-frequency global structure or aberrant high-frequency local cues). FFC’s spectral Transformer explicitly operates on these frequency components, leveraging FFT/iFFT for efficient global modeling, and it fuses outputs with spatial features to reinforce complementary cues. This fusion strengthens anomaly signatures by aligning local and global deviations, enabling robust detection of anomalies that perturb normalcy across scales.

**Computational Complexity of FFC.** We analyze the parameter count and FLOPs of the FFC block by decomposing it into four components: local-to-local ($Y^{l\to l}$), global-to-global ($Y^{g\to g}$), and two cross-branch paths ($Y^{l\to g},Y^{g\to l}$). Let $C_{1}$, $C_{2}$ denote the input/output channels, H×W the spatial resolution, *K* the kernel size, and α∈[0,1] the global channel ratio. For FFC, each component contributes the following:

- Local-to-local:
  $$\#\mathrm{Params}={\left(1-\alpha \right)}^{2}C_{1}C_{2}K^{2},\;\mathrm{FLOPs}={\left(1-\alpha \right)}^{2}C_{1}C_{2}K^{2}HW.$$
- Global-to-global:
  $$\#\mathrm{Params}=\frac{\alpha^{2}}{2}C_{2}\left(C_{1}+3C_{2}\right),\;\mathrm{FLOPs}=\frac{\alpha^{2}}{2}C_{1}C_{2}HW+\frac{13\alpha^{2}}{16}C_{2}^{2}HW.$$
- Cross-branch (l→g, g→l):
  $$\#\mathrm{Params}=\alpha \left(1-\alpha \right)C_{1}C_{2}K^{2},\;\mathrm{FLOPs}=\alpha \left(1-\alpha \right)C_{1}C_{2}K^{2}HW.$$

Table 1 shows that FFC introduces global context modeling with manageable overhead, offering a strong trade-off between efficiency and representational capacity. A key strength of FFC lies in its ability to model global dependencies using only small spatial kernels, such as 1×1, thanks to the spectral branch. This provides an efficient alternative to traditional convolutions when large receptive fields are required.

### 3.4. Hidden Space Anomaly Simulation (HSAS)

Generalization is a fundamental property of neural networks, referring to their ability to apply knowledge learned from training data to new, unseen data after training is complete. However, as previously discussed, for the most primitive reconstruction-based methods [36], the overly strong generalization capability of neural networks can negatively impact the anomaly detection task.

To address this overgeneralization phenomenon, recent approaches have replaced pure reconstruction with denoising. Specifically, these methods simulate anomalies by adding noise to images or manually designing anomalies [10,19,37] while retaining the original reconstruction objective within the autoencoder framework. The input *X* is altered to $X^{\prime }$, thereby transforming the network’s loss function from Equation (1) to Equation (2). Nevertheless, the main limitation of such methods is that manually designed anomalies heavily depend on specific datasets and often differ significantly from real-world anomalies. As a result, some current methods attempt to introduce noise at the hidden feature level to overcome the limitations of pixel-level anomaly simulation. For example, SimpleNet [11] simulates anomalies by adding Gaussian noise to the feature level, while UniAD [7] simulates anomalies through masking parts of the tokens. Simulating anomalies at the feature level significantly enhances the network’s ability to reconstruct an anomaly-free appearance when faced with real-world anomalies.

In our proposed method, we introduce a strategy that simultaneously simulates anomaly features at both the latent and pixel levels. Specifically, at the feature level, we simulate anomalies by masking parts of the features using a 2D dropout layer to randomly drop out certain feature maps. We elaborate on this specific process in detail through the pseudocode provided in Algorithm 1. Given an input feature tensor $x\in R^{H\times W\times C}$, where *H*, *W*, and *C* denote the height, width, and number of channels, respectively, and a dropout probability p∈[0,1], the algorithm generates an anomaly-simulated feature tensor $y\in R^{H\times W\times C}$. The process begins by initializing an output tensor *y* to zero. A channel-wise binary mask $m\in {\left\{0,1\right\}}^{C}$ is then generated, where each element $m_{c}$ corresponds to whether the *c*-th channel should be dropped ($m_{c}=0$) or retained ($m_{c}=1$). This decision is made by sampling a random variable $r_{c}∼U\left(0,1\right)$ for each channel; if $r_{c}\leq p$, the channel is dropped ($m_{c}=0$); otherwise, it is retained ($m_{c}=1$). For each spatial position (h,w)∈{1,…,H}×{1,…,W}, the masking operation is applied such that $y_{h,w,c}=m_{c}\cdot x_{h,w,c}$. This ensures that if a channel is dropped, all its corresponding spatial features are set to zero, thereby simulating an anomaly in the hidden space. This approach allows us to effectively simulate anomalies by randomly masking parts of the feature maps, thereby enhancing the robustness and generalization of our model.
**Algorithm 1** Hidden Space Anomaly Simulation via Explicit 2D Channel-wise Dropout**Require:** Input feature tensor $x\in R^{H\times W\times C}$, dropout probability p∈[0,1]**Ensure:** Anomaly-simulated feature tensor $y\in R^{H\times W\times C}$
  1:Initialize the output tensor $y\leftarrow 0^{H\times W\times C}$  2:Generate the channel-wise binary mask $m\in {\left\{0,1\right\}}^{C}$  3:**for** each channel index c=1 to *C* **do**  4:   Sample a random variable $r_{c}∼U\left(0,1\right)$  5:   Set the mask: $m_{c}\leftarrow \{\begin{array}{cc} 0, & \mathrm{if}\;r_{c}\leq p\\ 1, & \mathrm{otherwise} \end{array}$  6:   **for** each spatial position (h,w)∈{1,…,H}×{1,…,W} **do**  7:     Apply masking: $y_{h,w,c}\leftarrow m_{c}\cdot x_{h,w,c}$  8:   **end for**  9:**end for**10:**return** *y*

Dropout was originally designed to prevent overfitting in neural networks by randomly deactivating neurons, thereby forcing the model to learn more distributed feature representations rather than relying on specific neuron pathways. This approach enhances the model’s robustness when encountering new data. However, in our method, we repurpose dropout to prevent the network from reconstructing both normal and anomalous patterns. The decoder receives the simulated defective features, which have been processed with dropout in the latent space, and it is trained using Equation (2) as the loss function.

## 4. Experiments

### 4.1. Dataset

Our experiments predominantly utilized the widely used MVTec Anomaly Detection dataset [38] and VisA dataset [39] for both anomaly detection and localization tasks. Similar to standard anomaly detection tasks, the training sets contain only images of normal samples, while the test sets include both normal and anomalous samples, along with their corresponding segmentation masks.

MVTec AD [38] is a widely recognized dataset for anomaly detection that consists of a total of 5354 images, with the training set containing 3629 normal samples. The testing set includes not only normal samples but also encompasses a variety of anomalies ranging from scratches, dents, colored spots, and cracks to combined defects. It contains 15 categories, including 10 object categories (bottle, cable, capsule, hazelnut, metal_nut, pill, screw, toothbrush, transistor, zipper) and 5 texture categories (carpet, grid, leather, tile, wood).

VisA [39] is a recent industrial anomaly detection dataset that comprises a total of 12 categories and 10,821 high-resolution images. Of these, 9621 normal samples are used to form the training set, while the remaining 1200 images constitute the test set. These 12 subsets can be categorized into three broad groups based on the characteristics of the objects. The first group comprises single-instance objects in a single image, such as Cashew, Chewing Gum, Fryum, and Pipe Fryum, and the second group consists of multiple instances in a single image, including Capsules, Candles, Macaroni1, and Macaroni2. The remaining are four subsets of printed circuit boards (PCBs) with intricate designs. Anomalous images include surface defects such as scratches, dents, colored spots, and cracks, as well as structural defects, such as misplacements or missing components.

Both the MVTec AD and VisA datasets are designed with real anomalies reserved for testing, while the training data include only normal samples. This setup inherently evaluates a model’s ability to generalize to unseen real-world defects, as the training phase does not expose the model to actual anomaly patterns.

### 4.2. Evaluation Metrics

Standard anomaly detection tasks typically use the Area Under the Receiver Operating Characteristic Curve (AUROC) as the primary evaluation metric. The AUROC is determined by plotting the True Positive Rate (TPR) against the False Positive Rate (FPR) at various threshold settings. It provides a comprehensive assessment of model performance across all possible classification thresholds, making it an invaluable tool for evaluating binary classifiers [40]. However, in scenarios with class imbalance, the AUROC may not accurately reflect the model’s performance [40]. Therefore, we employ the Average Precision (AP) [41] to further assess the model’s pixel-level anomaly detection capability. The AP is calculated as the weighted mean of precisions at each threshold, with the weights being the increase in recall from the previous threshold. Specifically, AP summarizes a precision–recall curve as the weighted mean of precision achieved when the recall increases. In many practical applications, anomalies can appear as connected components of various sizes. The Area Under Per-Region Overlap (AUPRO) [42] addresses this by balancing the consideration of these different-sized components, ensuring that the model performs well not only on large-scale anomalies but also on small-scale, subtle defects. The AUPRO involves iterative calculations of the overlap between predictions and the ground truth, focusing particularly on the region within a 30% false positive rate threshold. This approach offers a more detailed evaluation of anomaly localization, providing insights into how well the model can identify and localize anomalies within specific regions of interest.

### 4.3. Implementation Details

We followed the popular DRAEM [10] method for anomaly simulation, which utilizes an additional dataset, the Describable Textures Dataset [43]. All our experiments were conducted on a single NVIDIA RTX 3090 GPU (NVIDIA Corporation, Santa Clara, CA, USA). with a total batch size of 8 and training for 20,000 iterations. Consistency in the input data is maintained by normalizing the input images using the mean and standard deviation values obtained from the ImageNet dataset [6], followed by resizing to a uniform size of 256 × 256 pixels.

### 4.4. Main Results

**Quantitative results.** We report the image-wise AUROC for the image-level anomaly detection task in Table 2. Our approach achieves 100% discrimination accuracy across multiple categories and leads in average performance compared to current state-of-the-art methods, demonstrating its ability to accurately distinguish defective items of various materials and appearances.

For pixel-level outcomes, we report the results in Table 3 and Table 4. On average, our method excels in both pixel-level AUROC and pixel-level AP, achieving top scores across multiple categories. Specifically, our method achieves improvements of 0.4% and 1.2% on the AUROC and AP metrics, respectively.

Regarding region-wise performance, we compare using the AUPRO metric, as detailed in Table 5. Since AUPRO considers regional overlaps rather than pixel-level comparisons, it treats anomalies of any size equally. Our method surpasses state-of-the-art performance in over half of the categories, showcasing consistent performance across different sizes and shapes of anomalous regions.

In Table 6 and Table 7, we provide a quantitative comparison of our FFC-AD method against recent state-of-the-art anomaly detection methods using the AUROC metric at both the image level and pixel level. Our method achieves comparable performance on the image-wise AUROC metric. This is primarily due to other methods, such as those reported in [17,18], utilizing a WideResNet-50 as their feature extractor, whereas our approach employs a ResNet-18 with FFC layers. Despite the significant disparity in the number of parameters, our method remains competitive. On the pixel-wise AUROC metric, our method significantly outperforms other approaches, demonstrating its superior localization capabilities.

Table 8 shows that our method consistently achieves the best localization accuracy, surpassing the previous best-performing method by 3.3% in terms of average AP. Table 9 focuses on the AUPRO metric, which evaluates region-wise overlaps rather than pixel-level comparisons, thereby treating anomalies of any size equally. This consistent performance enhancement highlights the capability of our FFC-AD method in accurately identifying and localizing defect areas, regardless of their complexity or scale.

Our method achieves state-of-the-art performance across different metrics on multiple datasets, spanning diverse defect types and object/texture classes. RD [17], RD++ [18], and ViTAD [24] adopt a feature reconstruction paradigm that directly compares features from the input and reconstructed images. While effective at capturing high-level discrepancies, the high-dimensional nature of the feature space and the lack of explicit spatial cues limit their performance in fine-grained localization, leading to lower AP and AUPRO scores.

Diffusion-based approaches such as DiAD [20] and DDAD [22] introduce pretrained feature extractors to guide the reverse denoising process. However, the pixel-level fidelity of diffusion-generated outputs remains a challenge. These methods often struggle with accurately reconstructing small or fine-grained anomalies, thereby affecting both classification and localization precision. DRAEM [10] and DeSTSeg [19] leverage pixel-level pseudo-anomaly generation and segmentation-specific architectural designs. These techniques lead to competitive localization accuracy but rely heavily on carefully crafted training augmentations and specialized modules. SimpleNet [11] focuses on efficient anomaly simulation in the feature space. Although it performs well in distinguishing normal and anomalous samples, its lack of explicit pixel-level reconstruction or segmentation mechanisms limits its localization capability. RealNet [21] simulates anomalies at the image level, providing a more intuitive modeling of visual defects. However, the absence of a dedicated segmentation network restricts its ability to localize defects precisely. The consistent results across these categories of our FFC-AD demonstrate strong cross-category robustness and generalization to unseen anomalies.

**Figure 3:**
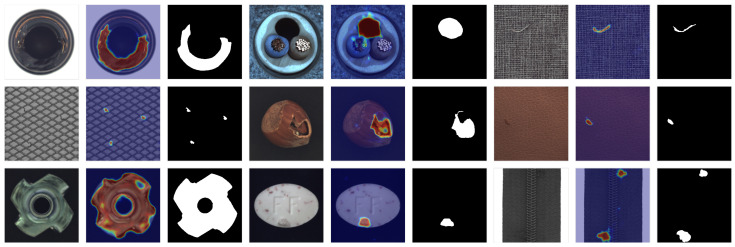
Qualitative visualized results for anomaly segmentation on the MVTec AD dataset. The sampled image, anomaly map, and ground truth are shown.

**Figure 4:**
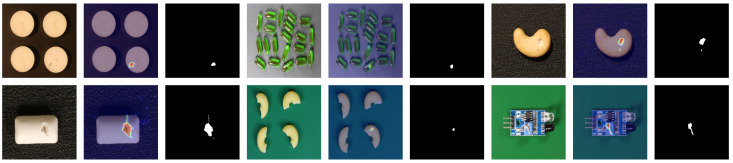
Qualitative visualized results for anomaly segmentation on the VisA dataset. The sampled image, anomaly map, and ground truth are shown.

**Qualitative results.** To further evaluate the effectiveness of our proposed method, we conduct qualitative experiments to demonstrate the anomaly localization performance. Figure 3 and Figure 4 illustrate the visualization results of our approach on the MVTec AD and VisA datasets, respectively. As can be seen from the figures, our method accurately distinguishes various defects across different categories. For large defects, our method provides sharp and compact edge localization. For small defects, our method achieves precise defect localization with no extraneous false detections. The consistent performance across different datasets demonstrates the generalization capability and versatility of our approach.

**Figure 5:**
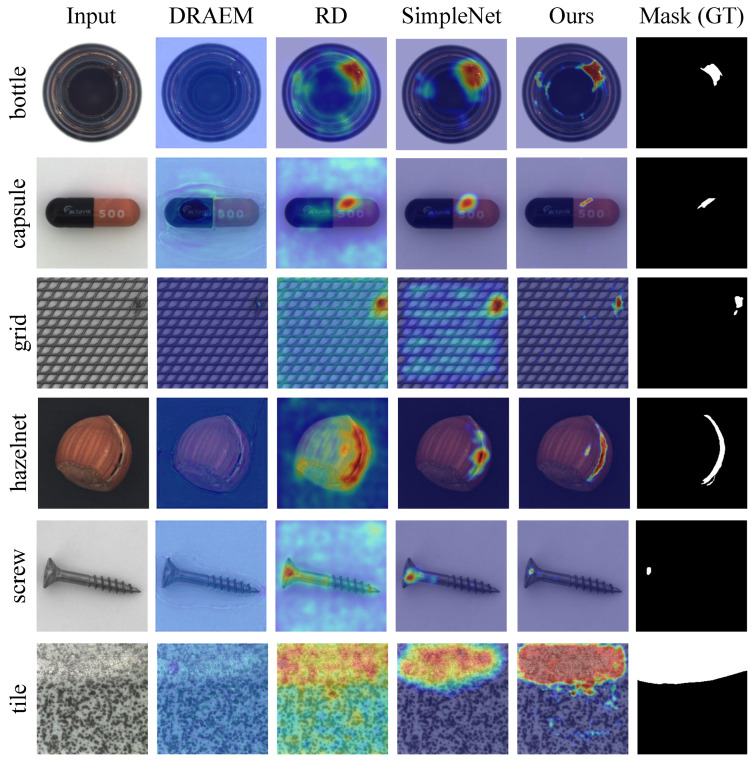
Qualitative comparison results on the MVTec AD dataset. The sampled image, anomaly map, and ground truth are shown.

Figure 5 presents a visual comparison of the results on the same sample using different methods. It can be observed that DRAEM [10] performs poorly on low-resolution images, failing to effectively identify abnormal regions. In contrast, RD [17] demonstrates a better capability in detecting various types of anomalies, with abnormal areas being more accurately localized. However, RD often suffers from a high false positive rate. For instance, in the “bottle” and “tile” samples, the significant number of false alarms could lead to unnecessary alerts in practical applications. SimpleNet [11] enhances the model’s generalization by adding noise at the feature level. Nevertheless, due to the lack of explicit segmentation constraints, it struggles to precisely locate the boundaries of small anomalies, as seen in the “capsule” and “screw” samples, indicating room for improvement in its segmentation performance. Our method achieves the best segmentation for both texture and object categories, with accurate, shape-preserving outputs handling anomalies of all sizes. Notably, in the “grid” and “screw” samples, it significantly reduces false positives while accurately localizing anomalies, demonstrating superior performance. In summary, our method excels across diverse anomaly detection scenarios, with great practical application potential.

**Efficiency results.** Our proposed method achieves a remarkable balance between computational efficiency and performance, as evidenced by the results in Table 10. With only 30 M parameters and 40 G FLOPs, FFC-AD outperforms state-of-the-art methods in both detection and localization. The lightweight design of FFC-AD is attributed to its frequency-domain feature transformation, which reduces redundancy in spatial feature learning while maintaining discriminative power. This makes it particularly suitable for real-world industrial applications where both accuracy and resource constraints are critical considerations.

**False detection.** As illustrated in Table 11, the F1-score achieved by our method reflects a balanced improvement in both precision and recall, outperforming all competing approaches. This dual enhancement indicates that FFC-AD effectively addresses two critical challenges in anomaly detection: reducing false positives (FPs) while maintaining sensitivity to false negatives (FNs). The high precision score demonstrates the model’s ability to suppress FPs, which are often caused by texture ambiguities or reconstruction artifacts in conventional methods. Simultaneously, the superior recall score highlights the model’s robustness against FNs, particularly in detecting subtle or low-contrast anomalies. The balanced precision–recall trade-off further underscores the effectiveness of HSAS in mitigating overgeneralization, a common issue in reconstruction-based models. By simulating anomalies in the hidden space, HSAS prevents the model from reconstructing defects as normal patterns, thereby reducing FNs without compromising precision.

### 4.5. Ablation Studies

**Main architecture.** As shown in Table 12, we conducted ablation studies focusing on the contributions of each component in our FFC-AD. Compared to experiment 1, introducing the FFC encoder results in slight improvements to both metrics. The detection AUROC increases to 98.7%, indicating that the FFC encoder effectively enhances feature extraction capabilities, contributing to better overall performance. In experiment 3, further incorporation of the FFC denoising autoencoder (FFC DAE) leads to additional gains in both detection and localization. The inclusion of the FFC DAE demonstrates its role in refining the anomaly detection process by reducing noise and enhancing robustness, thus improving both detection and localization accuracy. The final configuration integrates all three components, achieving the highest performance, with a detection AUROC of 98.9% and a localization AP of 77.0%. The introduction of Hidden Space Anomaly Simulation (HSAS) notably improves the model’s ability to accurately localize anomalies, suggesting its effectiveness in simulating and identifying anomalies in the hidden space. This hierarchical approach captures more nuanced spatial information, leading to enhanced anomaly detection and localization. In summary, the ablation study confirms that each component contributes positively to the overall performance of the FFC-AD method.

**FFC layer architecture.** To determine the optimal number of FFC layers within each FFC block, we conducted an ablation study. The results are summarized in Table 13. Incorporating two layers leads to significant improvements, achieving the highest performance metrics. This configuration is marked as the default entry due to its superior performance, suggesting that two layers provide an optimal balance between complexity and effectiveness. Increasing the number of layers to three slightly decreases performance compared to two layers, with a drop of 0.2% and 2.4% in detection AUROC and localization AP, respectively. This suggests that adding more layers beyond two may introduce unnecessary complexity without corresponding benefits.

As presented in Table 14, we further analyzed the impact of varying the ratio of the global part within the FFC block. With a global ratio of 0.25, the model achieves a detection AUROC of 98.3% and a localization AP of 75.3%. This lower ratio suggests that insufficient emphasis on the global context may limit the model’s ability to capture broader patterns. In defect detection tasks, although local details of defects are crucial, a certain degree of global context helps in understanding the overall scene where the defect is located, such as the relationship between the defect and the surrounding normal structure. Thus, an overly low global ratio may restrict the model’s capacity to capture broader patterns, making it difficult to accurately identify defects that are related to the overall scene. In contrast, increasing the global ratio to 0.75 also leads to a slight decline in performance. Particularly in defect detection tasks, most defects occupy only a small number of pixels. If the global ratio is too large, the model will allocate more computing resources and attention to the global information, resulting in insufficient extraction and analysis of the subtle local features of defects, which, in turn, affects the accuracy of detection and localization. In summary, the ablation study on the global-to-local ratio highlights that a balanced ratio of 0.5 provides the best overall performance. This configuration ensures that the model effectively captures both local and global features, leading to enhanced detection and localization capabilities.

**HSAS architecture.** We conducted ablation studies to investigate the impact of different dropout probabilities and types on the performance of the Hidden Space Anomaly Simulation (HSAS) module. The results are summarized in Table 15. We first evaluated the effect of varying dropout probabilities within the 2D dropout configuration. Increasing the dropout probability from 0.01 to 0.04 slightly improves the localization AP to 76.2%. This suggests that the mild neuron deactivation begins to disrupt overreliance on specific pathways but remains limited in promoting distributed feature learning, as the sparse deactivation leaves most original patterns intact, hindering the decoder’s ability to distinguish simulated defects. Setting the dropout probability to 0.1 yields the highest performance detection AUROC of 98.9%. This suggests that a moderate level of dropout effectively enhances feature robustness without overfitting, which is consistent with the method’s goal of repurposing dropout to disrupt co-adaptation between normal and anomalous pattern reconstruction. Further increasing the dropout probability to 0.2 leads to a slight decrease in both detection AUROC and localization AP. Excessive neuron deactivation impairs the decoder’s access to meaningful latent space features, as the aggressive randomness undermines the integrity of simulated defective patterns. This suggests that dropout probabilities must be calibrated to preserve the structural integrity of simulated defects while achieving the desired regularization effect.

We also compared the performance using 1D dropout with the probability of 0.1. While this configuration matches the detection performance of the optimal 2D dropout setting, it falls slightly short in localization accuracy. This discrepancy can be attributed to the inherent structural differences between the two dropout mechanisms; 2D dropout operates on spatial dimensions, preserving local contextual relationships while introducing randomness, which aligns with the spatial nature of anomalous patterns in our task. In contrast, 1D dropout, which acts along the feature channel axis, disrupts inter-channel dependencies but fails to adequately model the spatial continuity critical for precise localization. This result reinforces that 2D dropout is more effective for capturing spatial dependencies in anomaly detection tasks, particularly when precise localization of defective regions is required.

**Optimizer choice.** To evaluate the impact of different optimizers on the performance of anomaly detection, we conducted an ablation study comparing AdamW and SGD (Stochastic Gradient Descent). The results are summarized in Table 16. AdamW is known for its adaptive learning rates for different parameters, which can help in faster convergence and better handling of sparse gradients. With the SGD optimizer, the model reaches a higher performance. SGD’s simplicity and effectiveness in navigating the loss landscape with momentum often lead to better generalization and higher accuracy, especially in tasks requiring precise localization.

## 5. More Discussion

### 5.1. Limitations

**Network Depth Sensitivity.** Although the FFC backbone enables global information capture at shallow layers, the overall performance and generalization of the model may still be influenced by the depth of the network. Deeper networks potentially offer more expressive features but also increase the training complexity and risk of overfitting.

**Dataset Dependence.** Our method is primarily evaluated on industrial inspection benchmarks (MVTec AD and VisA). For instance, DRAEM [10] has been shown to be highly effective in industrial domains but may not generalize equally well to datasets with high semantic variability or unstructured noise. Validating the robustness of our method across a broader range of datasets remains an open question.

### 5.2. Generalizations

Our proposed HSAS regularization is implemented in the latent space by simulating anomalies through dropout-based perturbations. Unlike image-space corruptions, this approach is domain-agnostic, as it encourages the network to infer complete normal patterns from partial representations without requiring explicit spatial priors. As a result, the HSAS framework can be readily extended to other detection tasks where anomalies manifest as deviations from dominant patterns, including domains such as medical imaging or satellite surveillance.

However, for pixel-wise anomaly segmentation tasks, a key challenge arises: latent-space manipulations may lack precise spatial correspondence with the image domain. Hence, segmentation accuracy in new domains may hinge on the availability of domain-specific supervision or adaptations in the decoder structure to better preserve localization. Moreover, while the FFC structure enhances global context modeling, its inductive bias is still rooted in frequency decomposition. Domains with high spatial variance or where anomalies are characterized by fine-grained local texture irregularities (e.g., skin lesions, microcalcifications) may require complementary mechanisms to fully leverage FFC’s potential.

### 5.3. Future Directions

**Adaptation for Video Anomaly Detection.** Extending our FFC-AD to 4D representations could unlock new capabilities in video-based surveillance or dynamic industrial processes, where temporal coherence is critical.

**Cross-Domain Validation.** Further experiments on datasets from medical imaging, aerial inspection, or biometric security could help evaluate the robustness of FFC-AD under diverse visual statistics and anomaly types.

**Real-Time and Lightweight Architectures.** Optimizing the FFC modules for edge deployment or distilling their representations into efficient alternatives could extend the applicability of our framework in practical scenarios.

**Latent Space Regularization.** Beyond HSAS, future work may explore structured latent corruptions (e.g., attention-guided masking, variational sampling) to further improve anomaly discrimination and reduce overfitting.

## 6. Conclusions

In this work, we propose a novel framework, FFC-AD, which leverages the Fast Fourier Transform to address the limitations of CNN-based feature extractors that often lack global information. Furthermore, we introduce a strategy for simulating anomalies in the latent space, which allows the reconstruction model to overcome the overgeneralizing problem effectively. We demonstrate that our model significantly improves over previous state-of-the-art methods through comprehensive experiments on the challenging anomaly detection benchmarks. We achieve an increase of 2.2% in AP and 2.7% in AUPRO on the MVTec AD dataset and an increase of 3.3% in AP and 2.3% in AUPRO on the VisA dataset. These empirical results validate our proposed method’s effectiveness and highlight its potential for broader applications in anomaly detection.

## Figures and Tables

**Table 1:** Theoretical and empirical comparison of standard convolution and the FFC block. $C_{1},C_{2}$ denote input/output channels, H×W is the spatial resolution, *K* is the kernel size, and α is the global channel ratio. The ResNet-50 backbone is used for empirical measurement.

Metric	Standard Conv	FFC Block (α=0.5)
#Params (theoretical)	$C_{1}C_{2}K^{2}$	$\left(1-\alpha^{2}\right)C_{1}C_{2}K^{2}+\alpha^{2}C_{2}\left(\frac{1}{2}C_{1}+\frac{3}{2}C_{2}\right)$
FLOPs (theoretical)	$C_{1}C_{2}K^{2}HW$	$\left(1-\alpha^{2}\right)C_{1}C_{2}K^{2}HW+\alpha^{2}C_{2}HW\left(\frac{1}{2}C_{1}+\frac{13}{16}C_{2}\right)$
#Params (ResNet-50)	25.6 M	27.7 M
FLOPs (ResNet-50)	4.1 G	4.5 G

**Table 2:** Comparison of image-wise AUROC with state-of-the-art works on the MVTec AD dataset. Grey background is specifically used to highlight our results for clear differentiation. The best results are denoted in **bold**.

Method	DRAEM [10]	RD [17]	RD++ [18]	DeSTSeg [19]	SimpleNet [11]	RealNet [21]	DiAD [20]	DDAD [22]	GLAD [23]	ViTAD [24]	Ours
bottle	96.9	99.6	99.8	**100.0**	98.5	98.7	97.8	98.6	100.0	100.0	**100.0**
cable	93.4	81.9	94.5	93.0	99.9	63.3	85.9	**100.0**	**100.0**	98.8	95.7
capsule	96.1	98.2	97.6	91.3	100.0	51.0	97.9	98.3	99.5	95.8	97.2
carpet	96.2	98.4	97.4	93.6	100.0	97.3	91.3	100.0	100.0	99.4	**100.0**
grid	**100.0**	97.8	99.1	99.8	99.4	97.4	99.7	95.6	98.9	99.6	**100.0**
hazelnut	**100.0**	**100.0**	93.4	99.7	100.0	99.4	96.6	96.5	100.0	99.6	99.6
leather	**100.0**	**100.0**	**100.0**	**100.0**	99.9	**100.0**	97.4	99.5	99.7	**100.0**	**100.0**
metal_nut	99.4	59.9	**100.0**	**100.0**	99.4	63.4	94.6	99.2	98.1	99.9	98.8
pill	96.7	97.0	**97.6**	88.9	99.7	63.3	94.7	99.8	99.8	96.2	96.2
screw	**98.7**	98.3	**98.7**	86.4	100.0	72.7	72.4	99.5	100.0	91.2	98.4
tile	**100.0**	97.9	99.9	**100.0**	98.0	98.7	93.0	97.1	96.6	99.9	**100.0**
toothbrush	99.7	35.8	96.7	93.9	96.2	85.0	95.0	96.5	94.9	**100.0**	**98.9**
transistor	94.2	93.7	95.4	99.5	91.1	73.2	99.0	100.0	100.0	98.5	**100.0**
wood	99.1	99.4	99.3	99.2	99.7	**99.6**	93.5	99.8	99.4	98.9	99.0
zipper	99.7	99.5	68.2	99.4	99.8	80.6	71.8	99.8	98.1	97.7	**100.0**
Average	98.0	90.5	95.8	96.3	98.8	82.9	92.0	98.7	**98.9**	98.4	**98.9**

**Table 3:** Comparison of pixel-wise AUROC with state-of-the-art works on the MVTec AD dataset. The grey background is specifically used to highlight our results for clear differentiation. The best results are denoted in **bold**.

Method	DRAEM [10]	RD [17]	RD++ [18]	DeSTSeg [19]	SimpleNet [11]	RealNet [21]	DiAD [20]	DDAD [22]	GLAD [23]	ViTAD [24]	Ours
bottle	98.9	97.8	98.2	95.7	80.8	72.5	93.5	98.7	**99.4**	98.8	99.3
cable	97.7	84.9	93.7	92.2	76.8	59.8	90.8	**99.4**	**99.4**	95.4	95.5
capsule	99.0	98.8	98.8	86	93.4	51.8	90.3	98.5	**99.7**	98.1	99.0
carpet	99.1	99.0	98.8	96.1	95.4	87.8	87.7	98.1	98.4	98.9	**99.2**
grid	98.9	99.2	99.1	99.3	38.2	81.0	85.7	93.0	97.7	98.6	**99.4**
hazelnut	99.0	98.6	98.7	97.9	92.0	70.2	95.3	92.6	99.1	98.9	**99.7**
leather	99.4	99.3	99.2	99.5	82.2	95.8	91.5	98.2	98.4	99.5	**99.6**
metal_nut	98.8	92.1	96.6	96.9	79.9	51.6	94.4	95.8	98.7	96.1	**99.0**
pill	98.1	97.5	98.2	89.5	94.1	51.1	93.0	98.3	**99.2**	98.7	98.2
screw	**99.5**	99.4	99.5	74.4	91.3	50.6	86.4	99.0	98.8	98.9	98.9
tile	96.4	95.2	96.3	98.9	75.0	91.9	76.2	99.0	97.6	96.5	**99.3**
toothbrush	99.0	97.3	99.1	97.5	94.4	72.3	89.7	**99.3**	**99.3**	99.1	99.2
transistor	96.0	85.6	89.5	76.6	75.8	57.5	98.1	98.7	**99.2**	93.7	97.8
wood	95.2	95.5	95.3	94.1	74.6	87.4	83.1	95.4	**98.1**	96.1	97.4
zipper	98.9	98.4	98.3	94.7	92.6	65.5	84.0	98.0	97.7	95.9	**99.4**
Average	98.3	95.9	97.3	92.6	82.4	69.8	89.3	97.5	**98.7**	97.5	**98.7**

**Table 4:** Comparison of pixel-wise AP with state-of-the-art works on MVTec AD. The best results are denoted in **bold**.

Method	DRAEM [10]	RD [17]	RD++ [18]	DeSTSeg [19]	SimpleNet [11]	RealNet [21]	DiAD [20]	DDAD [22]	GLAD [23]	ViTAD [24]	Ours
bottle	89.7	68	71	88	42.2	60.5	45.9	74.0	78.1	80.0	**90.1**
cable	62.4	25.9	37.2	**62.8**	32.4	29.4	27.2	42.8	39.9	41.0	50.1
capsule	42.7	44.2	45.4	**57.5**	36.5	23.3	16.2	36.0	60.0	41.6	54.8
carpet	63.8	57.7	52.1	72.3	59.7	73.0	16.9	80.6	75.6	61.1	**80.3**
grid	55.3	45.9	43.7	**65.9**	40.3	47.8	3.9	61.9	70.9	30.6	63.0
hazelnut	87.7	58.3	57.1	90.2	67.9	47.1	27.9	52.0	86.0	64.1	**92.5**
leather	69.0	38.2	39.7	**80.6**	66.4	73.8	6.4	71.1	68.2	51.3	77.0
metal_nut	91.4	50	74.0	91.2	41.6	37.6	73.7	34.5	45.4	74.2	**93.7**
pill	46.0	63.1	73.4	**83.5**	45.0	41.1	50.4	51.1	68.3	77.5	81.1
screw	**70.6**	41.3	41.7	49.2	89.2	9.2	3.1	92.8	89.7	33.2	**56.0**
tile	**96.9**	48.4	52.8	**95.9**	79.5	84.8	25.0	84.0	67.8	56.5	94.9
toothbrush	53.4	21.3	57.0	**74.5**	30.5	41.5	11.1	38.1	26.2	54.7	71.4
transistor	51.3	41.7	48.8	60.4	50.1	48.6	72.8	43.3	46.5	57.6	**85.1**
wood	80.8	49.4	50.2	**83.6**	65.9	77.0	15.0	67.0	78.9	60.8	78.4
zipper	72.0	52.4	50.8	80.8	50.5	55.0	13.5	57.2	54.9	43.6	**85.9**
Average	68.7	47.1	53.0	75.8	53.2	50.0	27.3	59.1	63.8	55.2	**77.0**

**Table 5:** Comparison of region-wise AUPRO with state-of-the-art works on the MVTec AD dataset. The best results are denoted in **bold**.

Method	DRAEM [10]	RD [17]	RD++ [18]	DeSTSeg [19]	SimpleNet [11]	RealNet [21]	DiAD [20]	DDAD [22]	GLAD [23]	ViTAD [24]	Ours
bottle	85.3	94.6	94.8	92.6	91.4	61.5	75.9	94.1	97.0	94.7	**97.5**
cable	86.7	76.8	86.4	70.9	93.6	29.1	64.4	97.0	97.6	88.9	88.0
capsule	91.8	**96.0**	95.7	59	96.6	18.8	54.2	96.0	97.2	92.2	95.8
carpet	85.6	95.6	94.5	94.2	80.9	82.3	65.5	91.8	96.7	94.3	**97.8**
grid	91.3	97.3	96.7	**97.8**	80.5	70.3	57.4	79.2	95.0	95.8	96.5
hazelnut	89.7	96.4	96.4	90.5	91.3	64.3	81.0	76.3	97.3	95.2	**98.0**
leather	87.2	97.8	97.4	98.8	91.0	93.9	77.9	89.9	93.5	98.0	**99.2**
metal_nut	88.7	87.5	93.4	93.2	93.1	31.2	53.9	89.4	96.1	92.9	94.6
pill	88.4	96	96.3	48.9	93.9	28.3	65.7	87.1	91.1	95.5	**96.5**
screw	90.5	97.1	**97.7**	50.1	90.8	17.0	55.4	91.1	96.3	93.6	95.7
tile	88.4	85.7	88.7	**98.0**	92.4	87.3	50.5	93.1	96.7	88.0	97.2
toothbrush	88.1	86.1	92.2	81.1	94.8	25.6	64.9	96.6	95.5	91.0	**93.6**
transistor	87.8	74.8	78.3	78.2	82.4	31.6	85.3	93.5	96.1	77.0	90.7
wood	91.5	91.6	90.3	94.8	92.3	85.4	58.0	90.8	92.0	88.6	**95.6**
zipper	88.1	95.2	94.5	90.9	94.2	42.0	55.5	92.8	90.5	89.4	**97.9**
Average	88.6	91.2	92.9	82.6	90.6	51.2	64.4	90.6	95.2	91.7	**95.6**

**Table 6:** Comparison of image-wise AUROC with state-of-the-art works on the VisA dataset. The best results are denoted in **bold**.

Method	DRAEM [10]	RD [17]	RD++ [18]	DeSTSeg [19]	SimpleNet [11]	RealNet [21]	DiAD [20]	DDAD [22]	GLAD [23]	ViTAD [24]	Ours
candle	93.2	91.8	92.7	89.8	95.6	53.6	**99.3**	99.7	98.9	90.0	93.6
capsules	74.6	81.0	79.5	**86.5**	87.7	81.6	66.7	100.0	99.1	80.2	81.7
cashew	96.4	93.3	93.5	86.6	94.1	89.5	**99.8**	94.6	98.2	87.6	95.9
chewinggum	94.9	95.5	96.2	96.8	98.9	89	97.6	98.5	98.8	95.2	98.5
fryum	93.4	95.8	95.7	92.3	96.5	80.1	**97.0**	100.0	99.0	94.3	92.1
macaroni1	95.7	95.2	92.8	93.5	92.3	69.9	**96.3**	97.6	99.7	85.3	86.3
macaroni2	37.8	85.6	**82.8**	72.2	78.8	59.0	56.0	89.9	100.0	77.2	76.4
pcb1	65.7	96.4	**96.3**	78.6	98.7	82.7	91.7	100.0	99.7	95.6	94.7
pcb2	89.2	97.3	**97.9**	82.8	98.8	87	89.9	99.9	99.9	90.0	96.7
pcb3	94.1	96.8	95.7	95.8	98.0	87.0	94	100.0	100.0	91.2	**97.8**
pcb4	98.4	**99.9**	**99.9**	98.0	99.0	92.1	99.8	99.9	99.9	98.9	99.7
pipe_fryum	98.9	**98.2**	94.1	95	98.8	79.1	97.7	97.6	98.5	97.6	97.9
Average	86.0	**93.9**	93.1	89	94.8	79.2	90.5	**98.1**	99.3	90.3	92.6

**Table 7:** Comparison of pixel-wise AUROC with state-of-the-art works on the VisA dataset. The best results are denoted in **bold**.

Method	DRAEM [10]	RD [17]	RD++ [18]	DeSTSeg [19]	SimpleNet [11]	RealNet [21]	DiAD [20]	DDAD [22]	GLAD [23]	ViTAD [24]	Ours
candle	95.5	99	**99.3**	74.5	97.9	53.5	80.1	97.9	93.6	96.1	99.0
capsules	98.6	**99.3**	99.1	95.7	99.0	74.6	94.3	99.7	99.8	98.3	98.4
cashew	79.1	92.3	95.9	84.4	99.1	53.5	89.1	95.6	97.9	98.3	**99.6**
chewinggum	97.7	98.7	98.8	97.5	98.2	82.2	68.5	97.1	99.4	97.7	98.9
fryum	90.4	97.0	97.2	67.0	95.1	57.0	97.5	95.1	96.5	97.5	95.0
macaroni1	48.7	**99.8**	99.7	73.3	99.5	54.7	55.2	98.4	99.8	98.6	**99.8**
macaroni2	83.9	99.6	99.5	62.8	96.3	53.5	56.8	98.7	99.8	98.1	**99.7**
pcb1	96.5	99.5	99.4	96.0	99.6	73.6	95.7	91.0	99.2	99.4	**99.6**
pcb2	81.5	97.7	98	94.4	98.6	72.2	82.2	97.4	97.9	98.0	**98.7**
pcb3	98.3	98.0	98	90.6	98.8	76.9	87.2	97.2	98.5	98.3	**98.6**
pcb4	96.0	97.7	97.1	94.4	98.1	68.6	95.4	96.8	98.3	99.0	**97.9**
pipe_fryum	90.4	99.1	98.9	86.8	99.2	64.1	98.6	96.2	99.2	99.5	**99.7**
Average	92.3	98.1	98.4	84.8	98.3	65.4	83.4	96.8	98.3	98.2	**98.7**

**Table 8:** Comparison of pixel-wise AP with state-of-the-art works on the VisA dataset. The best results are denoted in **bold**.

Method	DRAEM [10]	RD [17]	RD++ [18]	DeSTSeg [19]	SimpleNet [11]	RealNet [21]	DiAD [20]	DDAD [22]	GLAD [23]	ViTAD [24]	Ours
candle	33.5	19.1	25.5	**39.5**	7.6	8.8	1.0	17.1	22.7	17.1	43.7
capsules	28.8	**57.3**	56.8	38.7	21.2	34.2	8.0	67.4	49.4	31.1	37.8
cashew	26.0	44.9	56.3	48.2	68.0	25.4	45.6	37.6	43.3	62.6	**84.5**
chewinggum	34.8	58.4	58.8	**78.3**	39.7	63.8	15.6	27.5	65.7	58.5	45.5
fryum	44.5	48.1	49.9	47.6	40.6	32.4	**59.0**	39.5	34.0	47.2	46.1
macaroni1	48.7	23.6	**24.0**	15.2	1.5	14.6	0.0	8.3	17.1	7.1	22.4
macaroni2	2.3	**12.3**	9.6	10.3	0.4	6.8	0.0	16.0	20.4	3.5	9.1
pcb1	52.7	66.8	64.9	66.5	73.1	40.1	27.7	12.5	48.8	65.6	**75.2**
pcb2	0.8	21.1	25.5	19.1	7.2	13.9	4.4	13.9	9.0	12.5	**28.8**
pcb3	11.1	23.8	30.9	27.5	12.9	24.6	4.0	7.9	23.0	22.6	**35.4**
pcb4	27.3	30	27.4	**53.8**	13.9	37.2	9.9	43.2	38.8	42.2	39.9
pipe_fryum	21.5	55.1	54.9	74.3	64.1	48.5	55.1	43.8	56.9	65.1	**90.2**
Average	27.7	38.4	40.4	43.3	29.2	29.2	19.2	27.9	35.7	36.4	**46.6**

**Table 9:** Comparison of region-wise AUPRO with state-of-the-art works on the VisA dataset. The best results are denoted in **bold**.

Method	DRAEM [10]	RD [17]	RD++ [18]	DeSTSeg [19]	SimpleNet [11]	RealNet [21]	DiAD [20]	DDAD [22]	GLAD [23]	ViTAD [24]	Ours
candle	93.1	95.2	95.4	49.9	80.4	53.6	41.4	95.7	86.8	86.5	**96.3**
capsules	85.7	91.9	91.4	77.8	57.5	39.6	55.3	97.9	96.3	76.0	**93.0**
cashew	78.0	84.5	85.1	55.8	85.0	29.0	21.5	71.1	93.0	79.3	92.4
chewinggum	76.6	78.1	80.1	68.4	74.7	42.3	18.6	88.0	94.7	71.4	87.0
fryum	79.4	**93.1**	92	35.4	78.5	22.8	68.9	90.7	93.4	88.9	89.4
macaroni1	81.8	97.2	96.7	36.6	66.5	19.7	3.6	98.0	98.3	90.7	**98.7**
macaroni2	78.8	97.0	96.2	36.0	66.4	20.1	15.8	98.6	98.6	88.1	**98.5**
pcb1	75.1	**96.1**	95.5	68.6	79.0	27.3	56.5	86.1	91.8	89.2	92.5
pcb2	67.6	91.7	**91.7**	69.8	76.0	40.3	48.1	93.1	90.7	82.6	91.5
pcb3	66.9	93.6	93.3	61.2	82.7	40.9	41.8	94.3	95.5	88.0	**94.0**
pcb4	65.9	88.4	85.8	74.9	67.1	31.4	82.4	87.4	92.9	93.5	**88.5**
pipe_fryum	65.0	95.9	94.2	55.9	76.6	39.6	77.9	81.1	97.3	94.9	**96.0**
Average	76.2	91.9	91.4	57.5	74.2	33.9	44.3	90.2	94.1	85.8	**93.2**

**Table 10:** Comparison of efficiency with state-of-the-art works on the MVTec dataset. The detection (Det.) results are reported in terms of AUROC, and the localization (Loc.) results are reported in terms of AP. Model parameters are reported in millions (M, $10^{6}$), and FLOPs are reported in Giga (G, $10^{9}$). The grey background is specifically used to highlight our results for clear differentiation. The best results are denoted in **bold**.

Method	DRAEM [10]	RD [17]	RD++ [18]	DeSTSeg [19]	SimpleNet [11]	RealNet [21]	DiAD [20]	DDAD [22]	GLAD [23]	Ours
Det.	55.2	90.5	95.8	96.3	79.2	82.9	92.0	98.7	99.0	**98.9**
Loc.	3.1	47.1	53.0	75.8	24.0	50.0	27.3	59.1	63.8	**77.0**
Params	97	81	96	35	73	591	1331	158	1066	**30**
FLOPs	198	28	38	31	**18**	115	452	757	2127	40

**Table 11:** Comparison of pixel-wise AP, F1-max, and the corresponding precision and recall with state-of-the-art works on the MVTec AD dataset. The grey background is specifically used to highlight our results for clear differentiation. The best results are denoted in **bold**.

Method	RIAD [44]	DRAEM [10]	RD [17]	SimpleNet [11]	RD++ [18]	Ours
AP	48.2	68.4	47.1	24.0	53.0	**77.0**
F1	37.4	66.7	57.3	55.3	58.1	**69.7**
Precision	32.5	65.9	50.5	47.8	51.6	**70.6**
Recall	51.0	69.6	67.9	68.2	67.8	**69.3**

**Table 12:** Ablation studies on our main designs. The detection (Det.) results are reported in terms of AUROC, and the localization (Loc.) results are reported in terms of AP. The default entry is marked as gray. The best results are denoted in **bold**.

Exp.	FFC Encoder	FFC DAE	HSAS	Det.	Loc.
1	-	-	-	98.4	73.1
2	✓	-	-	98.7	74.9
3	✓	✓	-	98.8	75.6
4	✓	✓	✓	**98.9**	**77.0**

**Table 13:** Ablation studies on the FFC block layer number. The default entry is marked as gray. The best results are denoted in **bold**.

Layer	Det.	Loc.
1	98.3	75.3
2	**98.9**	**77.0**
3	98.8	74.1

**Table 14:** Ablation studies on the FFC block ratio. The default entry is marked as gray. The best results are denoted in **bold**.

Ratio	Det.	Loc.
0.25	98.3	75.3
0.5	**98.9**	**77.0**
0.75	98.7	74.6

**Table 15:** Ablation studies on the design of the HSAS. The default entry is marked as gray. The best results are denoted in **bold**.

Type	Prob.	Det.	Loc.
2D	0.01	98.7	75.3
2D	0.04	98.3	76.2
2D	0.1	**98.9**	**77.0**
2D	0.2	98.2	76.8
1D	0.1	**98.9**	76.7

**Table 16:** Ablation studies on the optimizer. The default entry is marked as gray. The best results are denoted in **bold**.

Type	Det.	Loc.
AdamW	98.8	75.6
SGD	**98.9**	**77.0**

## Data Availability

All experiments in this study were conducted using publicly available datasets, and no new data were generated.
